# Biophenolic Compounds Influence the In-Mouth Perceived Intensity of Virgin Olive Oil Flavours and Off-Flavours

**DOI:** 10.3390/molecules25081969

**Published:** 2020-04-23

**Authors:** Alessandro Genovese, Ferdinando Mondola, Antonello Paduano, Raffaele Sacchi

**Affiliations:** 1 Department of Agricultural Sciences, University of Naples Federico II, Via Università 100, 80055 Portici, Italy; fe.mondola@libero.it (F.M.); sacchi@unina.it (R.S.); 2 Department of Agricultural and Environmental Science, University of Bari, Via Amendola 165/A, 70126 Bari, Italy; antonello.paduano@uniba.it

**Keywords:** extra virgin olive oil, sensory analysis, phenolic compounds, virgin olive oil off-flavours, panel test, volatile compounds, SPME-GC/MS

## Abstract

In this study, the influence of phenolic compounds on the sensory scores attributed to extra virgin olive oil (EVOO) by panel test was investigated. Two model olive oils (MOOs) with identical concentrations of volatile compounds, differing only in the amount of biophenols (297 vs. 511 mg kg^−1^), were analysed by two official panels and by SPME-GC/MS. Six other MOOs set up by the two previous models were also tested and analysed. They were formulated separately with the addition of three off-flavours (‘rancid’, ‘winey–vinegary’ and ‘fusty–muddy’). While high levels of EVOO phenolic compounds did not produce any effect on the headspace concentration of volatile compounds, they did affect the scores of both positive and negative sensory attributes of EVOO, due to the well-known in-mouth interactions between EVOO phenols, saliva and volatile compounds. In particular, a decrease of about 39% in the positive fruity score was found in the presence of a higher concentration of phenols. Regarding EVOO off-flavours, the higher level of phenolic compounds decreased by about 23% the score of ‘fusty–muddy’ defect and increased the score of ‘winey–vinegary’ defect about 733%. No important effect of EVOO phenolics on the perceived intensity of the ‘rancid’ defect was found. These findings could be helpful in explaining some discrepancies of panel test responses observed during extra virgin olive oil shelf life.

## 1. Introduction

The quality of extra virgin olive oil (EVOO) is defined by several chemical indices (acidity, peroxide value, UV and alkyl esters), but the sensory perception of its flavour is the ultimate determinant [1,2,3]. Sensory assessment identifies mainly positive attributes and defects in the oil, and it is critical for the oil’s quality classification according to the International Olive Council [4] and the EU legislation [1]. In fact, to be classified as “extra virgin” (highest quality), olive oil must have the presence of ‘fruity’ notes in its flavour and the absence of any unpleasant sensations, also defined as defects or off-flavours. The four main frequent off-flavours of virgin olive oil are ‘musty’, ‘winey’, ‘fusty–muddy’ and ‘rancid’.

The positive attributes ‘bitterness’ and ‘pungency’ in-mouth are also evaluated in EVOO by sensory assessors and, although they are not considered important in the quality classification of olive oil, they are very desirable [3,5,6].

The bitterness and pungency are mainly related to the quali-quantitative presence of phenolic compounds in EVOO [7,8], characterised by different health functions [6]. Olive oil phenolic compounds comprise simple phenols, lignans ((+)-1-acetoxypinoresinol and (+)-1-pinoresinol), flavonoids (luteolin and apigenin) and hydroxyl-isochromans, but the most abundant are the secoiridoid derivatives of the glycosides oleuropein and ligstroside. Among these, secoiridoid derivatives of oleuropein such as the dialdehydic form of elenoic acid linked to hydroxytyrosol (3,4-DHPEA-EDA or ‘oleacein’,) and the aldehydic form of elenoic acid linked to hydroxytyrosol (3,4-DHPEA-EA or ‘oleuropeine aglycon’) have been reported to be as the main contributors to EVOO bitterness. In contrast, the dialdehydic form of elenoic acid linked to tyrosol (or ‘oleocanthal’, p-HPEA-EDA) has been reported to be as the main compound responsible for the pungency of EVOO, perceived typically at the back of the tongue [7,8]. Commonly, four categories of EVOO can be pointed to in relation to the phenolic content. A level of phenolics equal to or lower than 220 mg kg^−1^ corresponds to non-bitter oils or oils with almost imperceptible bitterness; slight bitterness corresponds to 220–340 mg kg^−1^; bitter oils have phenol contents ranging from 340 to 410 mg kg^−1^; and a phenol content higher than 410 mg kg^−1^ corresponds to quite bitter or very bitter oils [9]. The concentration range of phenolic compounds is very wide in EVOOs because these substances are mainly affected by the agronomic and technological conditions of EVOO production such as cultivar, ripening stage, geographic origin of olives, crushing, malaxation, etc. [9].

It has been reported that EVOO phenolic compound–aroma interactions can affect the release of EVOO aroma compounds in the presence of human saliva [10,11]. It has been hypothesised that the complex formed from the interaction between EVOO phenolics and proline-rich proteins could bind aroma compounds and consequently decrease their level during head-space analysis and during organoleptic assessment of olive oil. In particular, an in vivo study showed that 1-penten-3-one, *trans*-2-hexenal and esters had a lower release in the presence of higher levels of biophenols (about 600 mg kg^−1^) after swallowing 3.5 mL olive oil. In contrast, linalool and 1-hexanol had a longer persistence in the breath than other compounds [10]. Another in vitro study of EVOO with a low–medium level of phenolic compounds (about 300 mg kg^−1^) showed the lowest headspace release for some ethyl esters, acetates, alcohols and ketones [11]. Consequently, the sensory assessment of EVOO could be affected by the presence and content of phenolic compounds. This effect could influence the score given by panellists for EVOOs e.g., slight ‘fusty–muddy’ notes, mainly related to some ester compounds [12], would not be perceived in very bitter–pungent EVOOs because of a physicochemical trapping effect by phenolics and saliva on esters [10,11].

Therefore, the wide-ranging level of ‘bitter’ and ‘pungent’ taste in EVOOs makes the use of sensory assessment to classify EVOOs into categories a tool not exempt from risk.

Although the volatile and phenolic compounds in EVOO have been widely studied [7,9,13,14,15], to date, no study has aimed to verify the effect of biophenols on the sensory assessment of olive oil flavour and off-flavour.

Therefore, the aim of this work was to investigate the effect of EVOO phenolic compounds on the sensory scores of positive and negative attributes assessed by a panel test. For this purpose, two model olive oils (MOOs) with identical concentrations of volatile compounds, differing only in the amount of biophenols, were used. This allowed us to study the positive sensory attributes (‘fruity’ quality) in oils differing in phenolic compound content only, without major differences in EVOO flavour composition. Six other MOOs, created by the two previous models, were formulated with the addition of the ‘rancid’, ‘winey–vinegary’ and ‘fusty–muddy’ off-flavour olive oil references (supplied by IOOC) in order to verify the possible masking or salting out effects of EVOO biophenols on the perception of common defects. The sensory assessment of MOOs was made by two different official panels according to the EU legislation [1].

## 2. Results and Discussion

### 2.1. Quality Indices and Phenolic Compounds

Table 1 reports the free acidity, peroxide value, ultraviolet indices (K_232_, K_270_, ΔK) and phenolic compounds in the MOO samples. The free acidity, peroxide value, K_232_, K_270_ and ΔK of the two samples remained within the legal limits of the category of extra virgin olive oil [1], showing no difference between the two MOOs. Total phenolic compounds, determined by colorimetric measurement using Folin–Ciocalteau reagent, were 297.5 and 510.8 mg kg^−1^, respectively, in the MOO control sample with lowest concentration of phenolic compounds (MOO+P) and in the MOO sample with highest level of phenolic compounds (MOO++P). The addition of the phenolic extract increased the level of total phenolic compounds of about 71%. This increase varied for each phenolic compound e.g., the phenolic compound that increased the least was hydroxytyrosol (26%); in contrast, the ligstroside *p*-HPEA-EA showed the greatest increase (77%). These findings confirmed that the extractability of the phenolic compounds is linked to the different hydrophobicities of these compounds [16]. However, the average level of 3,4-DHPEA-EDA and 3,4-DHPEA-EA, responsible for EVOO bitterness and the level of p-HPEA-EDA responsible for pungency feeling [7,8], increased in the same way by about 60% and 62%, respectively.

### 2.2. Volatile Compounds

Table 2 shows the headspace concentration of volatile compounds in the MOO samples. As expected, the two MOO samples without the added defects had a lower number of volatile compounds than MOOs with the added defects. The volatile compounds that characterised MOOs without the addition of defects were 1-penten-3-one, 3-pentanone, hexanal, 1-penten-3-ol, *trans*-2-hexenal, hexyl acetate, cis-3-hexen-1-ol acetate, 1-hexanol, *cis*-3-hexen-1-ol and *trans*-2-hexen-1-ol. These compounds are typically found in EVOO and are generated by endogenous olive enzymes in the LOX pathway, starting from linoleic and linolenic acids [17]. Other volatile compounds present in MOO samples without the addition of the defects were ethanol and ethyl acetate. The presence of ethanol is a residual due to the use of ethanol for the recovery from EVOO of the phenolic compounds subsequently added to MOO samples. 

However, its sensory contribution was very modest since it was found at levels very close to its odour threshold of 30 mg kg^−1^ [12]. Ethyl acetate was found in all the samples at concentration levels below its odour threshold (0.94 mg kg^−1^ [12]). Therefore, it did not contribute to the aroma of the MOOs. Ethanol and ethyl acetate are generated through sugar fermentation by microorganisms found on olive fruits. When LOX plays a major role during EVOO production, the odour of these two volatile compounds is not expected to be defective. On the other hand, ethanol and ethyl acetate become defective when they are present at higher levels [17]. In all MOO samples, the volatile compounds derived from the lipoxygenase pathway had the same concentration level, except for hexanal and 3-pentanone, which showed elevated levels in MOO samples with the rancid defect. In fact, these two volatile compounds derive from the auto-oxidation of fatty acids and are typically found in rancid oils. As expected, the MOO samples with the addition of defects had a greater number of compounds that originated from excessive fermentations, amino acid conversion, mould activity or lipid oxidation phenomena than MOOs without defects [17]. Most of these off-flavours were also common among the defects [12]. It is important to mention that the aim of this work was to obtain slightly defective MOO samples, therefore, it is obvious that the number and the quantity of off-flavour volatile compounds were not the same as has been reported in literature for very defective olive oils [12].

**Figure 1:**
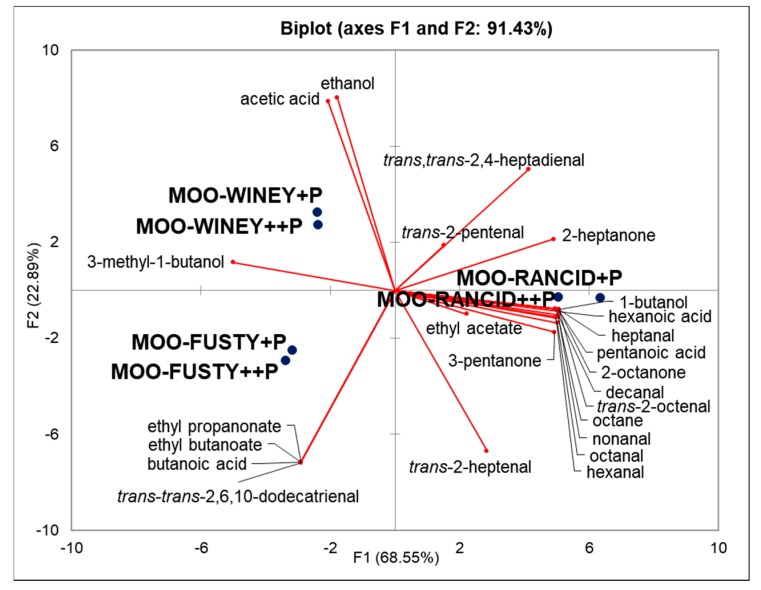
PCA based on SPME-GC/MS analysis of volatile compounds characterising MOO samples with the presence of sensory defects of ‘rancid’, ‘winey–vinegary’ and ‘fusty–muddy’. MOO+P code indicates the lowest level of phenolic compounds in model olive oil while MOO++P indicates the highest level.

In order to statistically explain the differences between defective MOOs in relation to the quality and quantity of off-flavour volatile compounds, a Principal Component Analysis (PCA) was also performed (Figure 1). For volatile compounds, those that did not present a significant difference among the MOO samples according to Tukey’s test (i.e., volatile compounds generated in the LOX pathway) were not considered (Table 2). PCA explained the 91.43% of variance using the first two principal components (PCs). The first PC clearly discriminated rancid MOO samples from fusty and winey MOO samples, while the second PC differentiated fusty MOO samples from winey MOO samples.

The volatile compounds predominant in ‘rancid’ MOOs, mainly produced by auto-oxidation of fatty acids, were hexanal, 3-pentanone, octane, *trans*-2-pentenal, 2-heptanone, heptanal, 1-butanol, 2-octanone, octanal, *trans*-2-heptenal, nonanal, decanal, *trans*-2-octenal, *trans*,*trans*-2-4-heptadienal, pentanoic acid and hexanoic acid. All these volatile compounds are usually linked to the rancidity of olive oil [15]. In fact, they are mainly characterised by rancid, oily and fatty sensory notes. Generally, aldehydes are the first compounds produced by the oxidation of unsaturated fatty acids, while acids appear at the end of the oxidative process due to the oxidation of the aldehydes previously formed [12].

The volatile compounds that characterised ‘winey’ and ‘fusty’ MOOs were acetic acid and ethanol for the first defect and *trans*, *trans*-2,6,10-dodecatrienal, ethyl propanoate, ethyl butanoate and butanoic acid for the second, while 3-methyl-1-butanol was significant for both defects. Both defects are due to microbial contamination of the olives. The winey defect is related to fermentation of *Lactobacillus* and acetic acid bacteria, while the fusty defect is linked to the Enterobacteriaceae genera *Aerobacter* and *Escherichia* at the beginning of olive storage, and the genera *Pseudomonas*, *Clostridium* and *Serratia* after extended olive storage [12].

It is evident that all volatile compounds analysed by SPME-GC/MS showed no differences between MOO+P and MOO++P samples (Table 2). These results were in agreement with previous studies [10,11] in which it was found that biophenolic compounds alone do not influence the headspace concentration of some C_5_ and C_6_ aroma compounds of EVOO. On the contrary, the studies demonstrated that phenolic compounds influenced the release of volatile compounds only in the presence of saliva. The first study was conducted in vitro with a retronasal aroma simulator (RAS) device, simulating mouth conditions with human saliva, while the second study was conducted in vivo by atmospheric pressure chemical ionisation–mass spectrometry (APCI/MS).

Therefore, it is correct to hypothesise that during olive oil tasting, i.e., when EVOO phenolic compounds enter into contact with saliva in the mouth, the sensory perception of EVOO is influenced by a different content of phenolic compounds. In order to verify this hypothesis, a sensorial analysis was performed to better understand whether and to what extent EVOO phenolic compounds affect the sensory perception of EVOO.

### 2.3. Sensory Analysis

Official sensory analysis includes a limited number of sensory attributes, namely four groups of defects and the positive attributes of olive ‘fruitiness’, ‘bitterness’ and ‘pungency’, of which only the first positive attribute classifies the category [4]. In addition, it should be highlighted that in the IOOC methodology panellists do not rate aroma and flavour attributes separately, but evaluate each perception as the whole olfactory–gustatory–tactile sensation. In fact, it is reported in the literature that the sensory description of EVOO is not impacted by the separate assessment of all sensations that form the flavour [18,19].

**Figure 2:**
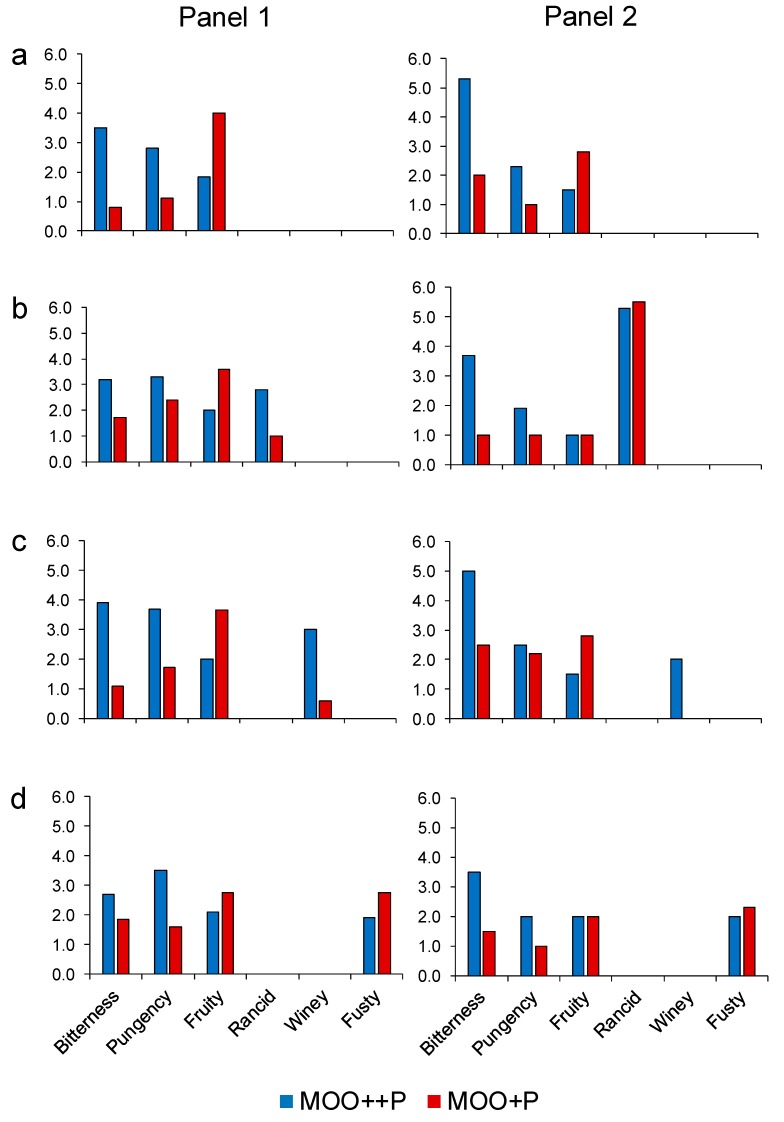
Sensory profiles of two panel tests obtained by analysing MOO+P in comparison to MOO++P samples without off-flavours (**a**) and with ‘rancid’ (**b**), ‘winey–vinegary’ (**c**) and ‘fusty–muddy’ (**d**) defects. MOO+P code indicates the lowest level of phenolic compounds in model olive oil while MOO++P is the highest level. Sensory attributes are expressed as median on an unstructured 0–10 scale.

Figure 2 shows the median value of the scores of positive and negative attributes in MOO samples without and with the addition of defects. As expected, the increase of phenolic compounds from 298 to 511 mg kg^−1^ (increase of 72%), previously verified by the Folin–Ciocalteau method (Table 1), determined an increase of the ‘bitterness’ and ‘pungency’ sensory attributes in all MOO++P samples. In particular, in the sample without the addition of defects (Figure 2a), the median values assigned by panels to MOO++P samples for these two sensory attributes were 3.5 and 5.3 for bitterness (for Panels 1 and 2, respectively) and 2.8 and 2.3 for pungency (for Panels 1 and 2, respectively). In contrast, the median values of MOO+P samples were 0.8 and 2 for ‘bitterness’ (for Panels 1 and 2, respectively) and 1.2 and 1 for ‘pungency’ sensory attributes (for Panels 1 and 2, respectively).

In MOO samples with the addition of defects, the ‘bitterness’ descriptor ranged from 2.7 to 5 in MOO++P samples and from 1 to 2.5 in the MOO+P samples. The ‘pungency’ descriptor showed a lower intensity than ‘bitterness’, and varied from 1.9 to 3.7 in MOO++P samples and from 1 to 2.4 in the MOO+P samples (Figure 2b–d).

In MOO samples without the addition of the defects, the highest level of biophenols (MOO++P) reduced the score of the sensory ‘fruity’ attribute by about 50% (1.9 vs. 4 and 1.5 vs. 2.8 for Panels 1 and 2, respectively) compared to MOO+P samples (Figure 2a), although the headspace concentration of the volatile compounds responsible for the ‘fruity’ note (1-penten-3-one, hexanal, *trans*-2-pentenal, 1-penten-3-ol, *trans*-2-hexenal, hexyl acetate, *cis*-3-hexen-1-ol acetate, 1-hexanol, *cis*-3-hexen-1-ol and *trans*-2-hexen-1-ol) showed no difference between MOO++P and MOO+P samples (Table 2).

These results were in agreement with the previous instrumental works on olive oil aroma [10,11].

The first work mentioned reported that EVOO phenolic compounds reduce the headspace concentrations of hexyl acetate, *cis*-3-hexen-1-ol acetate, 1-hexanol, *cis*-3-hexen-1-ol, *trans*-2-hexenal and 1-penten-3-one only in the presence of the saliva. The above compounds are responsible for the fruity descriptor as has been reported in literature for EVOO [13]. The second work studied the effect of phenolic compounds on the release of olive oil aroma compounds under in vivo conditions. Among eight monitored volatile compounds, 1-penten-3-one, *trans*-2-hexenal, *cis*-3-hexen-1-ol acetate, hexanal and 1-hexanol were included. The first three compounds had a lower release in the presence of higher levels of biophenols, while hexanol had a greater release. For hexanal, no important differences were found [10]. However, among the C_5_ and C_6_ volatile compounds that contribute to the fruity sensory note of EVOO, 1-penten-3-one and *trans*-2-hexenal are the main active odour volatile compounds. The former is important because of its low odour threshold, and the latter because of its usual high concentration in EVOO [20,21]. Therefore, it is possible to state that the score of positive ‘fruity’ descriptor was lower in MOO++P than MOO+P samples because the headspace concentration level mainly decreased for 1-penten-3-one and *trans*-2-hexenal in the presence of phenolic compounds and saliva. As a possible explanation, it has been reported that the formation of EVOO phenolic compound–proline-rich protein complexes could retain volatile compounds in the hydrophobic cavities and consequently decrease the concentration level in the headspace [10]. Saliva, increasing the stickiness of phenolic compounds to the oral surface, prolongs their retention even for long periods in the oral cavity despite a constant saliva flow [22], thus, easing the possible polyphenol–salivary proteins–aroma interactions. The effect of saliva–phenol interactions on aroma release has also been shown for red wines with both instrumental and sensory approaches [23,24,25]. In particular, sensory approaches showed how the intensities of ‘fruity’ and ‘floral’ aromas seemed to decrease when the level of polyphenols increased [26].

In the rancid MOO samples (Figure 2b), the scores assigned by panels to MOO++P samples for ‘fruity’ attribute were 2 and 1, respectively, for Panels 1 and 2. In MOO+P samples, the median values were 3.6 and 1, respectively, for Panels 1 and 2. The rancid descriptor showed for Panel 1 a higher value in the MOO++P sample (2.8) than the MOO+P sample (1), while Panel 2 showed higher intensity than Panel 1 but very few differences in the rancid attribute between the two MOO samples (5.3 vs. 5.5). Among the volatile compounds that impacted on ‘rancidity’ in MOO samples (hexanal, 3-pentanone, octane, *trans*-2-pentenal, 2-heptanone, heptanal, 1-butanol, 2-octanone, octanal, *trans*-2-heptenal, nonanal, decanal, *trans*-2-octenal, *trans*,*trans*-2-4-heptadienal, pentanoic acid and hexanoic acid) (Table 2, Figure 1), it has been reported in the literature that EVOO phenolics have no effect on hexanal and *trans*-2-pentenal [10,11]. Therefore, it is possible to hypothesise a similar behaviour to that of hexanal and *trans*-2-pentenal for the other aldehydes found in the ‘rancid’ MOO samples, such as heptanal, octanal, nonanal, *trans*-2-heptenal and *trans*-2-octenal.

In the ‘winey–vinegary’ MOO samples (Figure 2c), the higher level of phenolic compounds determined a lower intensity of the ‘fruity’ descriptor and a higher intensity of the ‘winey–vinegary’ defect. The median values assigned by panels to MOO++P samples for fruity attribute were 2 and 1.5, respectively, for Panels 1 and 2. In the MOO+P samples, the median values were 3.7 and 2.8, respectively, for Panels 1 and 2. The ‘winey–vinegary’ off-flavour was reported by Panel 1 to have a higher value in the MOO++P sample (3) than the MOO+P sample (0.6), while Panel 2 reported the ‘winey–vinegary’ attribute only in the MOO++P sample (2). The most involved volatile compounds for this defect are acetic acid and ethanol, followed by 3-methylbutanol (Table 2, Figure 1). Unfortunately, no published data were found regarding a possible influence of phenolic compounds on the aroma release of these compounds in the mouth or in in vitro systems in the presence of saliva.

Figure 2d shows the sensory results of MOO+P and MOO++P samples with the addition of the ‘fusty–muddy’ defect. In contrast to the previous defects, the fusty–muddy defect showed higher median values in MOO+P than MOO++P samples (2.8 vs. 1.9, Panel 1; 2.3 vs. 2.0 Panel 2). The most involved volatile compounds for this defect were ethyl propanoate, ethyl butanoate, butanoic acid, trans, trans-2,6,10-dodecatrienal and 3-methyl-1-butanol (Table 2, Figure 1). In the case of esters, it has been reported that phenolic compounds interacting with saliva are able to trap ethyl butyrate, ethyl isobutyrate and ethyl-2-methyl butyrate, reducing their in-mouth release [10,11]. Therefore, in the presence of a high level of phenolics, the impact of esters on the ‘fusty–muddy’ defect could decrease, making it less evident. Here again, no published data were found regarding the possible influence of olive oil phenolic compounds on the aroma release of *trans*, *trans*-2,6,10-dodecatrienal and 3-methyl-1-butanol.

The higher level of phenolic compounds also determined a lower intensity of the ‘fruity’ attribute descriptor, even if it was less than the other two off-flavours. In particular, the scores assigned by the panels to MOO++P samples for the ‘fruity’ attribute were 2.1 and 2, respectively, for Panels 1 and 2. In MOO+P samples, the median values were 2.8 and 2, respectively, for Panels 1 and 2.

**Figure 3:**
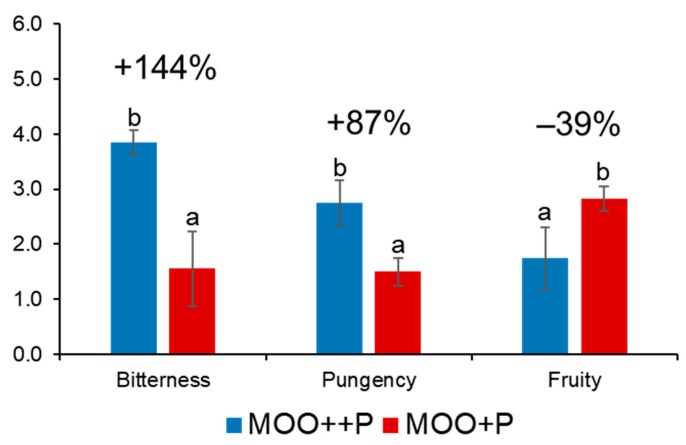
Sensory scores of ‘bitterness’, ‘pungency’ and ‘fruity’ attributes for all MOO samples obtained from the two panels. MOO+P code indicates the lowest level of phenolic compounds in model olive oil, while MOO++P is the highest level. Sensory attributes are expressed as the average of the median values obtained by using an unstructured 0–10 scale.

Finally, comparing the average of all MOO++P with all MOO+P samples, it is possible to state that 511 mg kg^−1^ of phenolic compounds in MOO++P samples increased the score of ‘bitterness’ and ‘pungency’ attributes by 144% and 87%, respectively, in comparison to the MOO+P samples, in which the phenolics were 297 mg kg^−1^. In contrast, the high level of phenolic compounds decreased the score of fruity attribute of 39% (Figure 3).

**Figure 4:**
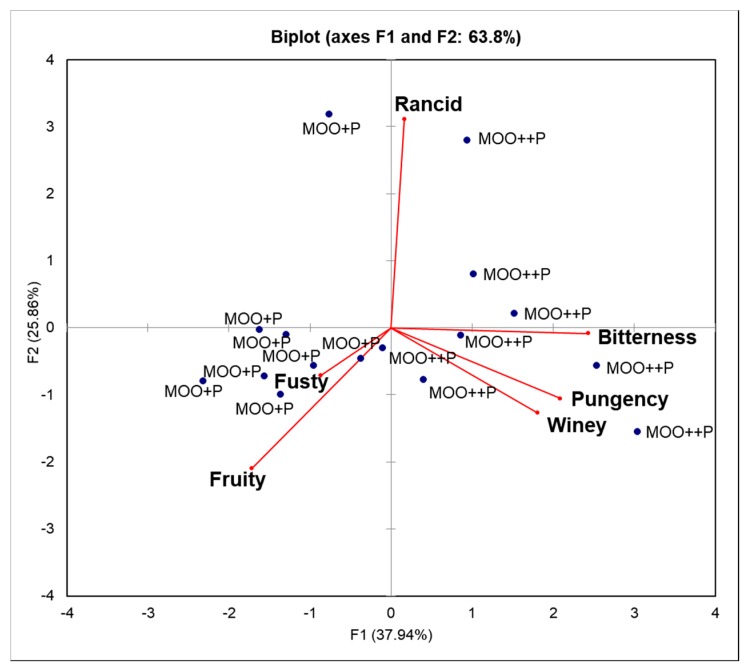
PCA based on sensory scores of the positive attributes of ‘fruity’, ‘bitterness’ and ‘pungency’ and the negative attributes of ‘rancid’, ‘winey–vinegary’ and ‘fusty–muddy’. MOO+P code indicates the lowest level of phenolic compounds in model olive oil, while MOO++P is the highest level. The sensory attributes were assessed by two different panels.

To statistically explain the differences between MOO++P and MOO+P samples in relation to positive and negative sensory attributes, a PCA was conducted on the sensory scores (Figure 4). The two main principal components explained 63.8% of the variance, with Principal Component 1 (F1) discriminating MOO++P and MOO+P samples. All MOO+P samples were characterised by ‘fruity’ and ‘fusty’ attributes. In contrast, the attributes ‘bitterness’, ‘pungency’ and ‘winey’ characterised all MOO++P samples. The ‘rancid’ off-flavour had little to no influence by the polyphenol content in the MOO. Therefore, a high level of phenolic compounds in EVOO could mask a ‘fusty–muddy’ off-flavour and enhance a ‘winey–vinegary’ off-flavour.

In conclusion, the sensory findings discussed here confirmed the hypothesis (one previously formulated in other studies, conducted by in vivo and in vitro instrumental approaches) that phenolic compounds, affecting the release of EVOO’s aroma compounds during its consumption, can influence the scores of perceived sensory attributes.

These results could be helpful in explaining some discrepancies of panel test responses commonly observed during extra virgin olive oil shelf life. In particular, in a 1 year old extra virgin olive oil, the in-mouth appearance of a ‘fusty–muddy’ defect could be due to the biophenol natural decrease over time (autoxidation). Therefore, this effect may change over time the original sensory classification made by the panel test from “extra virgin” to “virgin” olive oil.

Consequently, a high biophenol content (over 500–600 mg/kg) in bottled oils, apart from the possible use of EFSA nutritional claims on the label to inform consumers [5], could also ensure a sensory profile stability of bottled EVOO, avoiding the risk of slight sensory defects appearing during storage and preserving the EVOO legal classification.

## 3. Materials and Methods

### 3.1. Samples, Standards and Reagents

EVOO, named “Teti” and obtained from Rotondella (60%), Carpellese (30%) and Frantoio (10%) cultivars, was supplied by Torretta srl (Battipaglia, Salerno, Italy). For the production of Teti EVOO, the olives were harvested between 20 October and 30 November. Olives were washed and crushed using a disk crusher. The olive paste was then centrifuged using a three-phase low-volume water decanter. EVOO was placed into green glass bottles (500 mL) and stored in the dark at 19 °C to prevent oxidation until the moment of the chemical analysis, which was carried out at the fourth month of storage.

EVOO samples were characterised by the following quality indices: acidity 0.39 (±0.05), PV 6.40 (±0.1), K*_232_* 1.775 (±0.018), K*_270_* 0.121 (±0.001) and ΔK 0.005 (±0.001). The median of the fruity attribute (MF) was 5.2, while the median of each defect (MD) was equal to 0.

The composition of the biophenols was as follows: hydroxytyrosol 9.1 ± 0.1 mg kg^−1^, tyrosol 7.4 ± 0.2 mg kg^−1^, dialdehydic form of elenoic acid linked to hydroxytyrosol (3,4-DHPEA-EDA) 50.9 ± 0.6 mg kg^−1^, dialdehydic form of elenoic acid linked to tyrosol (*p*-HPEA-EDA) 47.9 ± 0.5 mg kg^−1^, pinoresinol and acetoxypinoresol (lignans) 30.1 ± 1.1 mg kg^−1^, aldehydic form of elenoic acid linked to hydroxytyrosol (3,4-DHPEA-EA) 45.7 ± 1.0 mg kg^−1^, aldehydic form of elenoic acid linked to tyrosol (*p*-HPEA-EA) 14.7 ± 0.4 mg kg^−1^, total phenolic compounds by HPLC 205.6 ± 1.0 mg kg^−1^ and by Folin–Ciocalteau method 348.4 ± 8.2 mg kg^−1^.

The refined olive oil (ROO) was supplied by Dorella Oleificio Candela srl (Castellamare di Stabia, Napoli, Italy). The International Olive Oil Council (IOOC, Madrid, Spain) supplied samples for each of the three defected reference oils, usually used in the process of training assessors to detect sensory defects. Each standard oil was characterised by one of the following off-flavours with its median of the defect (MD): ‘rancid’ (MD = 9.6), ‘winey–vinegary’ (MD = 5.6) and ‘fusty–muddy’ (MD = 6.9).

Hexanal (97%), *cis*-3-hexenylacetate (98%), ethyl acetate (99%), *trans*-2-hexenal (95%), 1-hexanol (98%), 1-penten-3-one (95%), octane (99.7%), 3-pentanone (99%), *trans*-2-pentenal (95%), 1-penten-3-ol (99%), 2-heptanone (99%), heptanal (95%), 3-methyl-1-butanol (99%), hexyl acetate (99%), 2-octanone (99.9%), octanal (99%), *cis*-3-hexen-1-ol (98%), nonanal (95%), *trans*-2-hexen-1-ol (96%), decanal (99%), *trans*-2-octenal (94%) *trans*, *trans*-2,4-heptadienal (97%) and hexanoic acid (99%) were supplied by Sigma–Aldrich (St. Louis, MO, USA). Acetic acid (99%) and *trans*-2-heptenal 98% were supplied by Fluka (Buchs, Switzerland). The reagents glacial acetic acid, diethyl ether and distilled water were used for the analysis and were supplied by Romil (Cambridge, UK). HPLC-grade methanol (>99.9% purity), hexane (>95%), Folin–Ciocalteu reagent, anhydrous sodium carbonate (>99.5%), caffeic acid (97%), ammonium acetate (0.4%) and sodium hydroxide were bought from Sigma–Aldrich (St. Louis, MO, USA). Potassium iodide was provided by AppliChem (Darmstadt, Germany). Phenolphthalein and starch were provided by Titolchimica s.p.a. (Rovigo, Italy). Food-grade ethyl alcohol (96%) was supplied by Selex S.p.A. (Trezzano sul Naviglio, Milano, Italy).

### 3.2. Sample Preparation

To study the effect of phenolic compounds on the sensory scores of the panel test, two MOOs were set up with identical volatile compound concentrations but with different concentrations of polyphenols. Teti EVOO was used as the control sample with the lowest concentration of phenolic compounds (encoded MOO+P). The MOO with the highest level of phenolic compounds (encoded MOO++P) was obtained by adding to EVOO Teti ROO phenolic compounds extracted from the same Teti EVOO in order to obtain a MOO sample with about a double concentration of phenolic compounds compared to MOO+P. A ROO without phenolics was also added to the control sample (MOO+P), which was then subjected to the same protocol as MOO++P. In order to understand the possible effect of phenolic compounds on EVOO off-flavours, six other MOOs were prepared by adding a separate aliquot of each off-flavour IOOC olive oil reference (‘rancid’, ‘winey–vinegary’ and ‘fusty–muddy’) to the previous MOOs (MOO+P and MOO++P). Finally, a total of four MOO samples with the lowest concentration of phenolic compounds (MOO+P) were built, one without off-flavour and the remaining three with one off-flavour each: one ‘rancid’, another one with the off-flavour ‘winey–vinegary’ and the last one with off-flavour ‘fusty–muddy’. The other four MOOs were built at highest concentration of phenolic compounds (MOO++P) without and with the same three off-flavour additions, for a total of eight samples. MOOs were stored before sensory and SPME-GC/MS analyses in room conditions (19 °C), avoiding light exposure and high temperatures in order to prevent oxidation, and were used within one month of their preparation.

#### 3.2.1. Preparation of MOO Samples with the Addition of Virgin Olive Oil Phenolic Compounds

MOOs were prepared according to Genovese et al. [10,11]. The phenolic extract was obtained from Teti EVOO. An aliquot of the oil sample (50 g) was dissolved in hexane (100 mL). A subsequent extraction was carried out using a water/methanol mixture (40/60 v/v) in a separating funnel (500 mL), after having shaken it vigorously for 15 min in a 500 mL bottle. This step was repeated twice using a total of 140 mL solvent. Subsequently, the hydro-alcoholic extract that was obtained was washed with hexane to remove any oil contamination and was centrifuged for 5 min at 3500 rpm (ALC International srl, PK-120, Milan, Italy). The organic phase was removed from the sample, and the hydro-alcoholic phase was collected in the flask and evaporated under a vacuum in a rotary evaporator at 35 °C (Heidolph VV 2000, Germany). The phenolic compounds were suspended using 10 mL ethyl alcohol (food-grade). A total of 1.6 kg of Teti EVOO was used to extract phenolics. This phase was repeated several times in order to obtain a total of 280 mL of biophenol extract in ethyl alcohol. The phenolic extract was subsequently concentrated up to a final volume of 40 mL using a rotary evaporator at 35 °C (Heidolph, VV 2000). The 40 mL phenolic extract was added to a flask with 170 g of ROO. The oil mixture was stirred and treated in an ultrasonic bath for 5 min. Ethanol was then evaporated in a vacuum evaporator (Heidolph VV 200) at 35 °C.

ROO with phenolic compounds was diluted in 1680 g of Teti EVOO in order to obtain MOO++P with a level of total phenolic compounds of 511.0 ± 9.3 mg kg^−1^ in order to reproduce a very bitter oil. Indeed, Beltrán et al. [9] reported that above mentioned phenolic compounds level is to be considered the level of a very bitter EVOO. This level of phenolic compounds was chosen because in in vivo experiments it was shown to affect the release of aromatic compounds of EVOO [10]. In the control sample (MOO+P), phenolic extract was not added, but the sample was subjected to the same protocol previously described for the addition of the phenolic compounds. Therefore, 40 mL ethanol food grade was added to a flask with 170 g of ROO. The mixture was then stirred and treated in an ultrasonic bath, followed by the evaporation of ethanol in a vacuum evaporator. Finally, ROO was diluted in 1680 g of Teti EVOO in order to obtain a MOO+P sample with a level of total phenolic compounds of 297.5 ± 8.7 mg kg^−1^. This level has been reported as the typical level found in bitter oil [9].

#### 3.2.2. Preparation of MOO+P and MOO++P Samples with the Addition of the Defected Reference Oils

The amount of ‘standard defect oil’ to be added was established by preliminary sensory tests in laboratory. The aim was to achieve a slight defect in each MOO. In order to build MOO samples with the ‘rancid’, ‘winey–vinegary’ and ‘fusty–muddy’ off-flavours, 1.71 g, 1.64 g and 0.74 g of ‘rancid’, ‘winey–vinegary’ and ‘fusty–muddy’ reference oils, respectively, were weighed and diluted in 410 g of each of the two MOO samples (MOO+P and MOO++P).

### 3.3. EVOO and MOO Analysis

#### 3.3.1. Free Acidity, Peroxide Value and Specific Ultraviolet Absorbance K232 and K270

The Teti EVOO and MOO samples were analysed to determine their acidity levels, peroxide value (PV), K*_232_*, K*_270_* and ΔK, according to the EU legislation [1]. Acidity was expressed as oleic acid percentage (%); PV was expressed as meq O_2_ kg^−1^ oil. For the analysis of spectrophotometric indices, an ultraviolet–visible UV-1601 spectrophotometer (Shimadzu, Kyoto, Japan) was used. All the analyses were performed in triplicate.

#### 3.3.2. Extraction and Analysis of Phenolic Compounds

The extraction and analysis of phenolic compounds were carried out according to Sacchi et al. [27]. The quantification of phenolic compounds was carried out using the Folin–Ciocalteau and HPLC methods. The phenolic compounds were analysed in both Teti EVOO and MOO samples to confirm the quantity and composition of the added phenolics in the MOOs. The analyses were performed in triplicate for each extraction.

#### 3.3.3. Extraction and Analysis of Volatile Compounds

Dynamic headspace (DH) SPME-GC/MS was used for the analysis of volatile compounds in Teti EVOO and MOO samples according to Romero et al., [28]. The SPME device (Supelco Co., Bellefonte, CA, USA) was equipped with a 50/30 μm thickness divinylbenzene/carboxen/polydimethylsiloxane (DVB/CAR/PDMS) fibre coated with a 1 cm length stationary phase. A 2 g aliquot of EVOO was added to a 20 mL vial with 10 μL of isobutyl acetate (Sigma–Aldrich, St. Louis, USA; 560.1 mg kg^−1^ in refined olive oil), which was used as the internal standard (IS). The vial was then closed with a polytetrafluoroethylene (PTFE) septum. The fibre was exposed for 40 min at 40 °C after 10 min at 40 °C for equilibration [21]. Volatile compounds were analysed by GC/MS according to Morales et al. [12]. A Shimadzu QP5050A GC/MS instrument (Kyoto, Japan) was used with a Supelcowax−10 capillary column (60 m, 0.32 mm i.d., 0.5 μm thickness; Supelco Co., Bellefonte, PA, USA). Thermal desorption of volatile compounds was carried out by putting the SPME fibre in the injector for 10 min. The temperature was set at 40 °C for 6 min, followed by an increase of 2 °C min^−1^ up to 200 °C, and was then held for 10 min. The injector was kept at 230 °C. Helium was used as a carrier gas (0.9 mL min^−1^). The peak areas were calculated using Lab solutions acquisition system (GCMS solutions version 1.20; Shimadzu) and were normalised with respect to the area of the internal standard peak. Compound identification was performed by comparing retention times and mass spectra obtained by analysing pure reference compounds in the same conditions. The identification was further confirmed by comparing mass spectra with those of the National Institute of Standards and Technology (NIST) database. Mass spectra were recorded at 70 eV. The source temperature was 200 °C and the interface temperature was 230 °C. Before use, the fibre was conditioned at 270 °C for 1 h for the analysis. A blank test was carried out before every run to prevent the release of undesirable compounds. All analyses were performed in triplicate.

#### 3.3.4. Sensory Analysis

EVOO assessment was performed by two different panels according to the method described in the European Commission Regulation and further amendments [1], one at the Laboratorio Chimico Merceologico of CCIAA (Camera di Commercio Industria Artigianato e Agricoltura) of Naples (Italy) and the other at the CCIAA of Salerno (Italy), each of them made up of ten trained panellists. They scored the descriptors on a normalised unstructured sheet (from 0 to 10) according to the IOOC/UE official method. Panel tests were made in ‘blind’ conditions, and only after they had been performed the identities of the MOO samples were revealed to panel leaders.

### 3.4. Statistical Analysis of Data

Significant differences among the different model systems were determined for each compound by one-way ANOVA statistical analysis. Tukey’s test was used to discriminate among the means of the variables. Differences with *p* < 0.05 were considered significant. Principal component analysis (PCA) was chosen as an exploratory technique to investigate the separation among groups of observations. Data elaboration was carried out using XLSTAT (version 2014.5.03), an add-in software package for Microsoft Excel (Addinsoft Corp., Paris, France).

## Figures and Tables

**Table 1:** Legal quality indices and phenolic compounds of MOO samples at two different level of EVOO phenolic compounds.

	MOO+P	MOO++P	Legal Limits
Quality indices			
Acidity	0.38 ± 0.05a	0.37 ± 0.02a	≤0.80
Peroxide value	6.4 ± 0.1a	6.3 ± 0.1a	≤20
K*_232_*	1.821 ± 0.050a	1.837 ± 0.012a	≤2.50
K*_270_*	0.120 ± 0.003a	0.128 ± 0.003a	≤0.22
ΔK	0.004 ± 0.001a	0.004 ± 0.001a	≤0.01
Phenolic compound			
Hydroxytyrosol	10.0 ± 0.4a	12.6 ± 0.2b	-
Tyrosol	7.4 ± 0.1a	11.6 ± 0.6b	-
3,4-DHPEA-EDA	48.3 ± 2.1a	72.1 ± 0.8b	-
*p*-HPEA-EDA	44.7 ± 2.0a	72.2 ± 0.6b	-
Lignans	29.2 ± 1.8a	46.2 ± 0.5b	-
3,4-DHPEA-EA	40.2 ± 0.8a	68.5 ± 0.3b	-
*p*-HPEA-EA	13.1 ± 0.9a	23.2 ± 0.0b	-
Total phenolics (HPLC)	192.8 ± 4.3a	306.4 ± 3.0b	-
Total phenolics (Folin–Ciocalteau)	297.5 ± 8.7a	510.8 ± 9.3b	-

Acidity is expressed as oleic acid equivalent. Peroxide value is expressed as meq O_2_ kg^−1^ oil. Phenolic compounds obtained by HPLC analysis are expressed as mg tyrosol kg^−1^ of oil. Total phenolics obtained by Folin–Ciocalteau essay are expressed as mg caffeic acid kg^−1^ of oil. 3,4-DHPEA-EDA: dialdehydic form of elenoic acid linked to hydroxytyrosol; *p*-HPEA-EDA: dialdehydic form of elenoic acid linked to tyrosol; 3,4-DHPEA-EA: aldehydic form of elenoic acid linked to hydroxytyrosol; *p*-HPEA-EA: aldehydic form of elenoic acid linked to tyrosol; lignans: sum of pinoresinol and acetoxypinoresinol. Values are the average of three replicates of analysis. Values followed by different letters are significantly different (*p* < 0.05).

**Table 2:** Volatile compounds, expressed as mg kg^−1^ ± standard deviation, in MOO samples without and with the addition of EVOO off-flavours at two different concentrations of EVOO phenolic compounds.

Compound	MOO		MOO with the Addition of VOO Off-Flavour					
			Rancid		Winey–Vinegary		Fusty–Muddy	
	MOO+P	MOO++P	MOO+P	MOO++P	MOO+P	MOO++P	MOO+P	MOO++P
octane	nf A	nf A	0.36 ± 0.02 aC	0.30 ± 0.01 aC	0.07 ± 0.02 aB	0.09 ± 0.00 aB	0.09 ± 0.00 aB	0.08 ± 0.00 aB
ethyl acetate	0.18 ± 0.07 aAB	0.22 ± 0.00 aA	0.25 ± 0.01 aB	0.45 ± 0.05 bC	0.12 ± 0.00 aAB	0.33 ± 0.01 bAB	0.10 ± 0.00 aA	0.35 ± 0.01 bBC
ethanol	30.08 ± 2.43 aA	31.89 ± 1.17 aA	32.6 ± 2.51 aA	34.97 ± 0.13 aA	43.31 ± 0.89 aB	44.97 ± 0.69 aB	34.28 ± 2.96 aAB	31.43 ± 1.82 aA
ethyl propanoate	nf A	nf A	nf A	nf A	nf A	nf A	0.01 ± 0.00 aB	0.01 ± 0.00 aB
3-pentanone	0.32 ± 0.00 aA	0.32 ± 0.03 aAB	0.40 ± 0.01 aB	0.36 ± 0.02 aB	0.29 ± 0.00 aA	0.28 ± 0.01 aA	0.31 ± 0.02 aA	0.29 ± 0.00 aAB
1-penten-3-one	0.24 ± 0.01 aA	0.23 ± 0.01 aA	0.26 ± 0.02 aA	0.23 ± 0.02 aA	0.28 ± 0.03 aA	0.25 ± 0.00 aA	0.24 ± 0.03 aA	0.24 ± 0.00 aA
ethyl butanoate	nf A	nf A	nf A	nf A	nf A	nf A	0.04 ± 0.00 aB	0.04 ± 0.00 aB
hexanal	2.23 ± 0.15 aA	2.24 ± 0.06 aA	3.12 ± 0.11 aB	2.87 ± 0.23 aB	2.27 ± 0.10 aA	2.25 ± 0.12 aA	2.38 ± 0.27 aA	2.23 ± 0.04 aA
*trans*-2-pentenal	nf A	nf A	0.09 ± 0.01 aB	0.09 ± 0.00 aC	0.11 ± 0.02 aB	0.07 ± 0.01 aBC	0.10 ± 0.01 aB	0.07 ± 0.01 aB
1-penten-3-ol	0.25 ± 0.03 aA	0.26 ± 0.03 aA	0.21 ± 0.02 aA	0.18 ± 0.01 aA	0.25 ± 0.01 aA	0.24 ± 0.01 aA	0.23 ± 0.00 aA	0.23 ± 0.03 aA
2-heptanone	nf A	nf A	0.38 ± 0.04 aC	0.38 ± 0.00 aC	0.11 ± 0.01 aB	0.15 ± 0.00 aB	nf A	nf A
heptanal	nf A	nf A	0.26 ± 0.01 aB	0.21 ± 0.02 aB	nf A	nf A	nf A	nf A
3-methyl-1-butanol	nf A	nf A	nf A	nf A	0.08 ± 0.00 aB	0.09 ± 0.01 aB	0.07 ± 0.01 aB	0.08 ± 0.00 aB
*trans*-2-hexenal	29.03 ± 1.84 aA	26.91 ± 2.17 aA	32.4 ± 0.79 aA	27.9 ± 1.98 aA	28.55 ± 0.81 aA	26.13 ± 3.63 aA	31.22 ± 2.44 aA	26.43 ± 1.50 aA
1-butanol	nf A	nf A	0.04 ± 0.00 aB	0.04 ± 0.00 aB	nf A	nf A	nf A	nf A
hexyl acetate	0.31 ± 0.02 aAB	0.28 ± 0.03 aA	0.34 ± 0.00 aB	0.30 ± 0.02 aA	0.26 ± 0.00 aA	0.25 ± 0.03 aA	0.31 ± 0.02 aAB	0.26 ± 0.00 aA
2-octanone	nf A	nf A	0.10 ± 0.00 aB	0.08 ± 0.01 aB	nf A	nf A	nf A	nf A
octanal	nf A	nf A	0.84 ± 0.04 aC	0.69 ± 0.05 aB	0.05 ± 0.01 aBC	0.05 ± 0.00 aA	0.1 ± 0.01 aB	0.11 ± 0.01 aA
*trans*, *trans*-2,6,10-dodecatrienal	nf A	nf A	nf A	nf A	nf A	nf A	0.12 ± 0.02 aB	0.13 ± 0.00 aB
*cis*-3-hexen-1-ol acetate	1.89 ± 0.17 aA	1.71 ± 0.20 aA	2.03 ± 0.05 aA	1.74 ± 0.18 aA	1.70 ± 0.11 aA	1.58 ± 0.25 aA	1.97 ± 0.21 aA	1.62 ± 0.07 aA
*trans*-2-heptenal	nf A	nf A	0.20 ± 0.01 aB	0.30 ± 0.03 aC	nf A	nf A	0.2 ± 0.01 aB	0.19 ± 0.01 aB
1-hexanol	2.58 ± 0.21 aA	2.36 ± 0.15 aA	2.89 ± 0.05 aA	2.39 ± 0.16 aA	2.66 ± 0.16 aA	2.42 ± 0.44 aA	2.84 ± 0.19 aA	2.34 ± 0.13 aA
*cis*-3-hexen-1-ol	3.04 ± 0.21 aA	2.81 ± 0.27 aA	3.39 ± 0.07 aA	2.82 ± 0.24 aA	3.20 ± 0.17 aA	2.92 ± 0.44 aA	3.37 ± 0.02 aA	2.76 ± 0.21 aA
nonanal	nf A	nf A	0.60 ± 0.03 aC	0.52 ± 0.05 aC	0.16 ± 0.01 aB	0.19 ± 0.03 aB	0.2 ± 0.02 aB	0.17 ± 0.00 aB
*trans*-2-hexen-1-ol	4.46 ± 0.32 aA	4.12 ± 0.34 aA	5.06 ± 0.16 aA	4.16 ± 0.36 aA	4.70 ± 0.26 aA	4.24 ± 0.69 aA	4.97 ± 0.31 aA	4.06 ± 0.26 aA
decanal	nf A	nf A	0.05 ± 0.00 aB	0.03 ± 0.00 aB	nf A	nf A	nf A	nf A
*trans*-2-octenal	nf A	nf A	0.06 ± 0.01 aB	0.06 ± 0.00 aB	nf A	nf A	nf A	nf A
*trans*, *trans*-2,4-heptadienal	nf A	nf A	0.08 ± 0.00 aC	0.08 ± 0.01 aC	0.06 ± 0.00 aB	0.05 ± 0.00 aB	nf A	nf A
acetic acid	nf A	nf A	nf A	nf A	1.22 ± 0.11 aB	0.83 ± 0.09 aB	nf A	nf A
butanoic acid	nf A	nf A	nf A	nf A	nf A	nf A	0.34 ± 0.01 aB	0.33 ± 0.01 aB
pentanoic acid	nf A	nf A	0.50 ± 0.02 aB	0.37 ± 0.05 aB	nf A	nf A	nf A	nf A
hexanoic acid	nf A	nf A	2.7 ± 0.11 aB	2.3 ± 0.09 aC	0.2 ± 0.07 aA	0.22 ± 0.04 aB	0.25 ± 0.07 aA	0.23 ± 0.03 aB

Values with different letters are significantly different (*p* < 0.05). Small letters indicate significant differences between MOO+P and MOO++P samples. Capital letters indicate significant differences among the samples without and with the addition of the defects. nf = Not found.
